# Genetic Predisposition and Infidelity: From Molecular Variation to Relationship Behavior—A Narrative Review

**DOI:** 10.3390/ijms27198510

**Published:** 2026-09-24

**Authors:** Ana Karina Zambrano

**Affiliations:** Centro de Investigación Genética y Genómica, Facultad de Ciencias de la Salud Eugenio Espejo, Universidad UTE, Quito 170129, Ecuador; anazambrano17@hotmail.com

**Keywords:** genetics, behavioral, extramarital relations, pair bond, dopamine D4, oxytocin, vasopressin, monoamine oxidase, gene–environment interaction

## Abstract

Trust and pair bonding are central to human social life, and their violation through infidelity carries a measurable psychological cost. Whether individual differences in such behavior are partly genetic remains contested, because the available evidence combines heterogeneous phenotypes with candidate gene designs of limited statistical power. This narrative review synthesizes genetic evidence on infidelity, pair bonding, responses to trust violation and indirect traits such as novelty seeking, grading it by evidentiary strength. Twin modeling of extrapair mating yields broad-sense heritability of approximately 62% in men and 40% in women, but this specific estimate has not been reproduced in a comparable sample and describes population variance, not individual causation. Reported associations for *DRD4*, *DAT1/SLC6A3*, *OXTR*, *AVPR1A*, and *MAO-A* are traced to well-characterized molecular substrates, whereas the behavioral associations are small, inconsistently replicated, and frequently drawn from adjacent rather than direct outcomes: meta-analysis does not support *OXTR* rs53576 as a correlate of close relationships, and *AVPR1A* RS3 findings reverse in direction across samples. The unresolved question is not whether genetic variation matters, but at what magnitude and through which measurable phenotype. Adequately powered, ancestrally diverse, and functionally validated studies are required before any predictive claim can be entertained.

## 1. Introduction

Trust is among the most fundamental attributes of individuals’ social lives, associated with numerous positive behaviors and interpersonal outcomes [1]. Betrayal is a profound sense of harm caused by the intentional acts or omissions of a trusted individual [2]. In its many forms, it encompasses infidelity, dishonesty, emotional or physical abuse, broken promises, and lying, among others [2,3]. The psychological consequences can include painful experiences, subsequent loss of trust, preoccupation, damaged self-esteem, anger, and self-doubt [1,2].

Betrayal has shadowed human civilization from its earliest recorded moments, from the assassination of Julius Caesar by Marcus Brutus [4] and the New Testament account of Judas Iscariot [5,6] to the sack of Constantinople in 1204 [7] and Benedict Arnold’s defection during the American Revolution [8]. These episodes illustrate the cultural salience of betrayal, but they are narrative rather than empirical.

Jennifer Freyd introduced the concept of betrayal trauma in 1994, referring to the harm inflicted by someone on whom the victim depends, creating a conflict between the need to acknowledge harm and the need to preserve attachment for survival [9]. Freyd and colleagues have explained that the victim often experiences betrayal blindness, in which awareness of the betrayal is suppressed to maintain the relationship and ensure safety; this dissociative process has been proposed to explain why some abused individuals do not recall trauma until later in life [9]. The concept implies that the human capacity for deep relational trust is inseparable from vulnerability to profound psychological injury.

Sexual infidelity is one of the most studied manifestations of interpersonal betrayal. Its definition varies by perspective; infidelity has been divided into sexual, emotional, combined, and internet-based forms [10]; broadly, it denotes covert emotional, sexual, or romantic behavior that violates the exclusivity rules established within a relationship [11]. Prevalence is difficult to estimate reliably because it depends on definition, recall, and disclosure [12]. A meta-analytic synthesis reported a lifetime prevalence of infidelity of 34% in men and 24% in women [13,14]. Nine successive waves of the United States General Social Survey, based on in-person interviews of independent probability samples of the adult population, showed that the lifetime prevalence of extramarital sex among ever-married adults declined from 17.8% in 2000 to 16.3% in 2016, while annual prevalence of extramarital sex remained stable (3%) [15]. Other reviews place lifetime estimates for married couples at roughly 20–35% [16]. This heterogeneity in measurement is itself a central obstacle to genetic research on infidelity.

The question of whether humans harbor innate tendencies toward betrayal, or whether such behavior is primarily shaped by environment, has attracted increasing scientific attention. Behavioral genetics, drawing on twin studies, has opened a window onto heritable contributions [17], and genome-wide association studies (GWAS) have implicated many common variants with small individual effects in behavioral traits [18]. The present article discusses the *DRD4*, *DAT1*, *OXTR*, *AVPR1A*, and *MAO-A* genes, which encode receptors, transporters, and enzymes, as candidate loci of mechanistic interest within a behavioral framework.

Candidate gene studies of behavioral phenotypes have typically been powered one to three orders of magnitude below what is required to detect the effect sizes for complex traits, and the published record shows the asymmetry characteristic of publication bias. For example, among 103 candidate gene-by-environment studies in psychiatry published between 2000 and 2009, 96% of novel reports were positive compared with 27% of replication attempts [19]. When 18 historic depression candidate genes, each studied ten or more times, were re-examined in a sample of up to 443,264 individuals, they proved no more associated with the phenotype than randomly chosen non-candidate genes [20]. More generally, complex behavioral traits are influenced by “very many genetic variants” [21]. This makes a large single-locus effect on a behavior as socially embedded as infidelity improbable a priori.

The aim of this narrative review is to synthesize the evidence linking genetic variation in five genes to betrayal-related behavior, to distinguish clearly between outcomes that measure infidelity itself and outcomes that measure adjacent constructs, and to identify the gaps that future research must close.

## 2. Literature Identification

PubMed, Scopus, and Web of Science were searched from database inception to 31 August 2026, combining phenotype terms (infidelity, betrayal, extrapair mating, trust violation, marital satisfaction, sexual partners, and extramarital sex) with terms for genetic predisposition (genetics, heritability, twin study, single nucleotide polymorphisms, genome-wide association studies, genetic variants, *DRD4*, *DAT1*, *OXTR*, *AVPR1A*, and *MAO-A).* Reference lists of retrieved articles and relevant reviews were screened manually. Eligible sources were peer-reviewed studies in humans published in English. Non-human studies were excluded, except where cited for mechanistic context, as were non-peer-reviewed sources, including conference abstracts, preprints, dissertations, editorials, and commentaries. Studies that reported no genetic, heritability, or functional measures were also excluded.

Four categories of outcomes were separated: (i) infidelity and extrapair mating; (ii) pair bonding and relationship stability; (iii) emotional and behavioral responses to a breach of trust; and (iv) indirect traits such as novelty seeking, impulsivity, aggression, and number of sexual partners.

## 3. Genetic Correlates of Betrayal and Infidelity

### 3.1. Twin-Based Heritability of Extrapair Mating

There is no evidence for the existence of a single “cheating gene”. Twin research indicates that infidelity has both environmental and heritable components [22]. A study of 7378 Finnish twins and their siblings estimated the genetic contribution to extrapair mating over a one-year period at approximately 40% in women and 62% in men [23]. According to the authors, this is a surprisingly high value, given that such behavior depends not only on the individual but on contextual factors as well [23]. The phenotype in that study was a proxy: participants were coded positive for extrapair mating if they reported more than one sexual partner in the preceding twelve months while in a relationship, a measure that captures the behavior indirectly and depends on truthful self-report [23]. Heritability is a population- and environment-specific variance ratio. It quantifies the proportion of observed variation attributable to genetic variation in that population under those conditions; it does not indicate what proportion of any individual’s behavior is caused by genes, and it implies neither biological determination nor predictability from genotype [24]. In the same analysis, most genetic variance was assigned to non-additive rather than additive components, and the confidence intervals for the individual variance components included zero, the authors noting limited power to distinguish the two [23]. A second twin study, of over 1600 UK female twin pairs, reported heritability of 41% for infidelity and 38% for lifetime number of sexual partners [22]. Both cohorts therefore indicate a heritable component, but they differ in population, sex composition and phenotype definition, and their estimates are not directly comparable.

The same authors also tested *AVPR1A* and *OXTR* for association with extrapair mating in that sample, and found a gene-based association for *AVPR1A* in women but not in men, and no significant association for *OXTR* in either sex [23].

### 3.2. Genome-Wide Evidence 

No GWAS has taken infidelity or betrayal as its phenotype. The most informative genome-wide data come from adjacent behaviors. In a combined sample of over one million individuals, genome-wide analyses examined risk tolerance, adventurousness, and risky behavior, including lifetime number of sexual partners as one of the risky behavior phenotypes (*n* = 370,711) [25]. SNP-based heritability was 0.128 for number of sexual partners [25]. Moreover, a genome-wide polygenic score predicted approximately 0.8% of the variance in number of sexual partners, and there was no evidence of enrichment among genes previously hypothesized to relate to risk tolerance [25]. A GWAS of same-sex sexual behavior in 477,522 individuals reached a comparable conclusion for a different sexual phenotype: the trait is highly polygenic, all tested variants together accounted for 8–25% of variation, and the authors stated explicitly that the results do not allow meaningful prediction of an individual’s behavior [26]. These results indicate the range of effect sizes to be expected for human sexual and risk-related behavior, and are consistent with a polygenic architecture.

### 3.3. Neurotransmitter Systems

Molecular studies have focused on variants in neurotransmitter systems involved in reward, attachment, and behavioral regulation. Candidate gene studies of behavioral traits such as reward sensitivity, impulse control, and novelty seeking have been documented in specific populations, some of them in small samples and under particular environmental conditions. For each locus, the molecular characterization and the behavioral associations attributed to it are considered separately below.

#### 3.3.1. The Dopamine System: *DRD4* and *DAT1*

The brain’s dopamine reward system plays a central role in reward and bonding [27]. Dopamine receptors are implicated in many biological processes (cognition, memory, and control of neuroendocrine signaling) [28]. Behavioral studies have accordingly implicated dopamine-related genes in sensation seeking and sexual behavior.

- ***DRD4***

The human dopamine receptor D4 (*DRD4)* gene mediates neuronal signaling through dopamine-dependent activation to regulate complex behavior and emotion [29]. It is located near the telomere of chromosome 11p15.5 and contains a 48-base-pair variable number tandem repeat (VNTR) in exon III ranging from 2 to 11 repeats [27]. At the molecular level, *DRD4* encodes a G-protein-coupled dopamine receptor that mediates neuronal signaling through Gαi/o proteins to inhibit adenylyl cyclase, lowering intracellular cyclic AMP (cAMP) and reducing protein kinase A (PKA) activity [29,30]. It participates in dopamine synthesis, release, and neuronal firing [31]. The exon III VNTR lies within the sequence encoding the receptor’s third cytoplasmic loop, which mediates G-protein coupling, and longer-repeat alleles could affect mRNA and protein expression levels [30,32].

In Chinese hamster ovary cells stably expressing the D4.2, D4.4, and D4.7 isoforms, dopamine inhibited forskolin-stimulated cAMP formation in a concentration-dependent manner for all three variants. The potency of dopamine was approximately two-fold lower for D4.7 (EC_50_~37 nM) than for D4.2 and D4.4 (EC_50_~16 nM); there was no consistent difference in the efficacy of inhibition, and deletion of the repeat did not abolish receptor function. The authors concluded that the polymorphic repeat causes only small changes in the receptor’s capacity to suppress cAMP production [33]. The measured effect is a rightward shift in agonist potency, and whether it translates into a measurable difference in postsynaptic signaling in the human brain has not been established. The proposed behavioral manifestation—heightened sensation seeking and disinhibition—may, in relational contexts, translate into elevated risk for infidelity and promiscuous sexual conduct [27,34,35,36].

At the meta-analytic level, pooled analyses of published adult non-psychiatric samples found no association between the exon III VNTR and approach-related personality traits. Although an association was initially observed for the *DRD4* C-521T polymorphism, this finding was possibly influenced by publication bias and could not be replicated in an independent sample selected for extreme extraversion scores. Moreover, the polymorphism was estimated to explain no more than approximately 3% of the phenotypic variance [37]. Similarly, a meta-analysis of the *DRD4* 7-repeat allele in relation to novelty seeking did not find robust evidence of an association, supporting the view that behavioral traits are likely shaped by multiple genetic variants of small effects [38].

A single study of 181 young adults reported associations between *DRD4* exon III VNTR and uncommitted sexual behavior. Carriers of at least one allele with seven or more repeats reported a higher categorical rate of promiscuous sexual behavior (45% vs. 24%, *p* = 0.018). The categorical difference in reported infidelity was in the same direction but did not reach statistical significance (50% vs. 22%, *p* = 0.14). They also reported an increase of more than 50% in the number of extrapair copulation partners among participants who had already reported being unfaithful (mean 1.79 vs. 1.14, *p* = 0.011); there was no difference in the total number of sexual partners [27]. This is a preliminary, single-sample association in a small ethnically heterogeneous student cohort; it is not evidence of an established causal pathway from *DRD4* genotype to betrayal.

- ***DAT1***

The dopamine transporter gene (*DAT1*, also known as *SLC6A3*, chromosome 5p15.3) contributes to dopaminergic processes including self-regulation, attention, and addiction [39]. It encodes a NA^+^/Cl^−^-dependent transport protein that is a regulator of dopaminergic signaling [40]. Its functional 40-base-pair VNTR lies in the 3′ untranslated region (3′UTR), a region influencing mRNA stability and transporter density [40]. In vitro, cells stably transfected with a construct carrying the 10-repeat allele displayed a maximal binding capacity approximately 50% greater than that of cells carrying the 9-repeat construct [40]. A small in vivo single-photon emission computed tomography study of 11 children with attention deficit/hyperactivity disorder found greater basal ganglia transporter density in 10-repeat homozygotes [41].

Across twelve positron emission tomography (PET) and single-photon emission computed tomography studies in 511 participants, the PET studies provided evidence that the 9-repeat allele, not the 10-repeat allele, was associated with increased striatal transporter availability (standardized mean difference 0.31, *p* < 0.001). The single-photon studies were highly heterogeneous [42].

Behaviorally, there are no reports of an association with infidelity; however, an adjacent phenotype study of approximately 2500 young adults reported that individuals homozygous for the 10-repeat allele had a significantly higher number of sexual partners than 9-repeat homozygotes, with the effect moderated by sex and social network characteristics [43].

The *DAT1* VNTR is therefore a functional polymorphism whose apparent direction of effect depends on the assay system, and inference from genotype to synaptic dopamine availability in the living human brain remains provisional.

#### 3.3.2. The Oxytocin System: *OXTR*

Oxytocin is a neuropeptide described originally for its role in parturition and lactation and subsequently recognized for its influence on social behaviors, including social motivation and approach [44]. Its neural substrates are best characterized in socially monogamous rodents, in which oxytocin receptor density in the nucleus accumbens and medial prefrontal cortex regulates partner preference formation [45,46].

The oxytocin receptor gene, *OXTR* (chromosome 3p25.3), has been linked to the development of the nervous system, social cognition, and behavioral functioning [47,48]. *OXTR* encodes a G-protein-coupled receptor that couples to Gαq/11, activating phospholipase C-β to hydrolyze phosphatidylinositol-4,5-bisphosphate (PIP_2_) into inositol-1,4,5-trisphosphate (IP_3_) and diacylglycerol (DAG); IP_3_ releases Ca^2+^ from endoplasmic reticulum stores while DAG activates protein kinase C, with additional MAPK/ERK and β-arrestin-dependent signaling in neurons [49]. Methylation of the *OXTR* CpG island is associated with receptor availability and social perception [50,51], and the common intronic variant rs53576 has been linked both to receptor expression and to genotype-dependent regulation of expression by DNA hydroxymethylation [50]. By contrast, rs9840864, rs2268494, rs7632287, and rs2268490 are haplotype-tagging markers selected for their coverage of linkage disequilibrium structure in association studies [47].

One study of 165 university students examined 10 *OXTR* SNPs in an iterated Prisoner’s Dilemma game in which participants believed they were interacting with a partner, although the partner was a pre-programmed computer strategy. After correction for multiple testing, C-rs9840864, T-rs2268494 was significantly associated with faster retaliation after betrayal; A-rs237887, C-rs2268490 with higher levels of post-betrayal satisfaction; and G-rs237887, C-rs2268490 with lower levels of post-betrayal satisfaction [47]. The sample was small, and the outcome measured was the response to a laboratory breach of trust rather than to betrayal within a relationship.

In a sample of 2309 individuals from the Twin and Offspring Study in Sweden and 1240 individuals from the Swedish Twin Study of Child and Adolescent Development, 12 *OXTR* SNPs were examined in relation to pair bonding and social behavior. The SNP rs7632287 was associated with pair bonding in women. Results suggested an association between specific SNPs and human pair bonding and other social behaviors [44]. The same SNP has been reported as associated with autism spectrum disorder in a meta-analysis, indicating pleiotropy at this locus [52].

The rs53576 polymorphism of *OXTR* has been linked to differences in social behaviors. The GG genotype at this locus has been associated with greater empathic accuracy, more secure attachment, and, in a study of 178 married couples, higher marital satisfaction relative to AA or AG genotypes [53]. However, these behavioral associations have not been consistently replicated in meta-analyses. A meta-analysis of rs53576 (*n* = 17,559) and rs2254298 (*n* = 13,547) across five domains (biology, personality, social behavior, psychopathology and autism) pooled 82 effect sizes and found that combined effects did not differ from zero in any domain or across all domains combined; the authors concluded that both SNPs failed to explain the social behavior analyzed [54]. A second meta-analysis separated the outcome domains: rs53576 was associated with general sociality (*n* = 4955, *p* = 0.02) but showed no association with close relationships (*n* = 5262, *p* = 0.64) [55].

Intranasal oxytocin administration in men in committed relationships led them to perceive their partners’ faces as more attractive than those of strangers, and oxytocin synchrony between partners has been reported to predict the durability of romantic bonds across early relationship stages [56,57]. Both studies were conducted in small samples, and the behavioral effects of exogenous oxytocin are themselves strongly context- and person-dependent [58].

#### 3.3.3. The Vasopressin System: *AVPR1A*

Arginine vasopressin is a neuropeptide, and its receptor gene, *AVPR1A* (chromosome 12q14.2), has been studied extensively in the context of pair bonding and monogamy [22,59,60]. *AVPR1A* encodes the V1a receptor, a Gαq/11-coupled receptor that activates the phospholipase C–Ca^2+^/PKC second-messenger system [61,62].

RS3 is a complex (CT)_4_-TT-(CT)_8_-(GT)_24_ polymorphism in the 5′-flanking promoter region; shorter RS3 alleles have been associated with reduced relative promoter activity and hence differences in receptor density [61]. RS3 was examined in relation to pair bonding, monogamy, and marital satisfaction in 552 Swedish twin pairs and their spouses [59]. Men carrying one or two copies of the 334 allele reported lower marital satisfaction and higher rates of marital crisis compared with non-carriers [59]. The difference in partner bonding scale score between men carrying at least one 334 allele and non-carriers corresponded to Cohen’s d = 0.27, rising to d = 0.38 for 334 homozygotes; 15% of males carrying no 334 allele reported marital crisis in the preceding year compared with 34% of 334 homozygotes. No association was found in women [59].

A study of over 1600 UK twins did not identify an association between *AVPR1A* and female infidelity or number of sexual partners, indicating that variation in these traits is not attributable to this locus alone [22].

A more recent study examined relationship maintenance in 70 newlywed couples genotyped for RS3; the couples reported on their marriage within three months of the wedding and on marital satisfaction every four months for three years. Carriers of the 334 allele reported fewer marital problems and less interest in romantic alternatives [60]. 

This directional reversal between a study of 552 established couples and one of 70 newlywed couples is consistent with the limitations of small candidate gene studies testing modest effects [60]. The authors proposed relational phase as a possible reconciliation, although this was not tested in the design [60].

Taken together, the *AVPR1A* findings are discordant in direction, in sex specificity, and in phenotype. 

#### 3.3.4. An Emerging Indirect Candidate Gene: *MAO-A*

*MAO-A* is an indirect candidate related to behavioral regulation. No published study has examined *MAO-A* in relation to infidelity or adjacent behavior; the available evidence concerns impulsivity, aggression, neural structure and function, and interaction with early adversity. Therefore, any extension to infidelity is an extrapolation from the aggression and impulsivity literature.

The monoamine oxidase A (*MAO-A*, chromosome Xp11.3) gene encodes a flavoenzyme anchored in the outer mitochondrial membrane that catalyzes the oxidative deamination of monoamine neurotransmitters such as serotonin, dopamine, and norepinephrine [63]. Enzyme abundance is set by a 30-base-pair upstream variable number tandem repeat (uVNTR) located approximately 1.2 kb upstream of the coding sequence. High-activity alleles (3.5 or 4 repeats) drive greater transcription than low-activity alleles (two, three, or five repeats), so the polymorphism scales the catabolic capacity of the enzyme and, consequently, synaptic monoamine availability [64]. In the original functional characterization, promoter–reporter fusion constructs transfected into three different cell types showed that alleles with 3.5 or 4 copies of the repeat were transcribed two- to ten-fold more efficiently than alleles with 3 or 5 copies, suggesting an optimal length for the regulatory region [65]. Low-activity genotypes have been associated with reduced behavioral inhibition, elevated impulsivity, and heightened reactivity to perceived social threat or rejection [66,67,68].

In a study combining neuroimaging with genotyping of *MAOA-H* (high-activity) and *MAOA-L* (low-activity) allele carriers in healthy individuals, *MAOA-L* carriers showed volume reductions in the bilateral amygdala, supragenual anterior cingulate, and subgenual anterior cingulate cortex, associated with a greater probability of impulsive violence in men [67]. A meta-analysis of gene–environment interaction studies supported the conclusion that low *MAO-A* activity is associated with antisocial and impulsive behavior among individuals with a history of childhood maltreatment [66]. The *MAO-A* by maltreatment interaction is among the more frequently replicated findings in the field, although the literature to which it belongs shows the novel-versus-replication asymmetry characteristic of publication bias, and a systematic review of the genetics of aggression more broadly reports the same pattern of small, inconsistently replicated candidate gene effects [19,69].

A summary of the candidate genes and studies is presented in Table 1 and Table 2. 

## 4. Molecular Signaling Pathways and the Functional Impact of Candidate Variants

Although the genes analyzed act in different neurochemical systems, their products converge on a small number of intracellular signaling modules (Table 1). *OXTR* and *AVPR1A* encode Gαq/11-coupled receptors and share the phospholipase C-β → IP_3_/Ca^2+^ and DAG/PKC cascade, whereas *DRD4* encodes a G-protein-coupled dopamine receptor and acts in the opposite direction by suppressing adenylyl cyclase and cAMP/PKA [30,32,49,61]. *DAT1* and *MAO-A* do not transduce a receptor signal; they set the amount and duration of monoamines available to these receptors [40,63].

Critically, the relevant variants previously described are concentrated in regulatory regions rather than in protein-coding sequences. The *DRD4* exon III VNTR modifies the receptor’s third intracellular loop and thereby its G-protein-coupling efficiency [30,32], although the measured consequence for cAMP signaling is a modest shift in agonist potency rather than a loss of function [33]; the *DAT1* 3′UTR VNTR tunes transporter mRNA stability and density [40], with a direction of effect that differs between in vitro and in vivo assays [40,42]; *OXTR* promoter and intronic variants influence methylation-sensitive transcription and receptor expression [50,71]; the *AVPR1A* RS3 repeat is a promoter microsatellite that scales transcription [61]; and the *MAO-A* uVNTR is a promoter element that sets enzymatic output [64,65]. A recurring feature of these variants is their effect on dosage, as they can modulate the amount of receptor or enzyme produced, or alter the efficiency of receptor signaling, generating quantitative differences in overall pathway activity.

Downstream, these influences converge: Gαq/11-driven Ca^2+^ and PKC signaling (*OXTR*, *AVPR1A*), cAMP/PKA tone (*DRD4*), and monoamine availability governed by *DAT1* and *MAO-A* [30,39,49,71]. From this convergence, it has been proposed that an individual becomes more vulnerable to betrayal when dopaminergic novelty drive outweighs oxytocinergic partner-specific bonding and prefrontal regulatory control is insufficient. No study has measured these pathways jointly in the same participants. The pathway-level account is therefore a framework for generating testable predictions, not a demonstrated mechanism. The individual links differ in evidential strength: the molecular steps, comprising receptor coupling and enzyme kinetics, are experimentally established, whereas the links running from pathway tone to behavior are inferred. The proposed opposition between dopaminergic novelty seeking and oxytocinergic bonding is a hypothesis at the behavioral level, although the opposition of the underlying second-messenger systems is experimentally established.

## 5. Neurochemical and Neural Mechanisms 

The genes described do not act directly on behavior; they shift the functional tone of neurotransmitter systems governing reward, bonding, and impulse control. In the dopaminergic pathway, genetic variants have been proposed to alter dopamine receptor sensitivity (*DRD4*) or dopamine availability (*DAT1*), with the possible consequence of a motivational state oriented toward novelty over familiarity; an individual with elevated novelty seeking may equally channel that disposition into varied non-sexual interests [27,72]. Oxytocin influences social bonding through hypothalamic and extended amygdala circuitry, modulating the rewarding value of familiar partners [73]. Variations in the *OXTR* gene may therefore be relevant to the victim’s emotional response to betrayal as well as to a perpetrator’s behavior [44,47,53,56,57,58]. The vasopressin signaling system, through *AVPR1A*, has been linked to receptor density in the ventral pallidum, a region implicated in consolidating partner preferences into lasting social memory [59]. The ventral pallidal mechanism is established in voles, whereas the corresponding human evidence is a small association with self-reported bonding [46,59].

Between genetic predisposition and behavioral expression lies the prefrontal cortex, the neural substrate of reasoning, impulse control, and moral cognition. In the context of betrayal, prefrontal function shapes the capacity to recognize a temptation, evaluate it against relational values, and inhibit the action before it occurs [72]. Prefrontal regulatory capacity may itself be under partial genetic influence: the low-activity *MAO-A* variants have been associated with reduced orbitofrontal and anterior cingulate volume and activity, which would be expected to affect the neural substrate of inhibitory control [66]. Figure 1 summarizes the proposed architecture from gene variants to behavior.

## 6. Genes, Environment, and Behavioral Regulation

One of the most persistent questions in the study of betrayal is whether humans are biologically predisposed to it or whether such behavior is shaped primarily by culture, environment, and conscious choice. The available evidence points to an interplay between genes and environment rather than dominance of either alone.

Biological impulses such as attraction, novelty seeking, or desire do not automatically translate into action. That translation depends substantially on the brain’s regulatory systems, particularly prefrontal cognitive control [72]. The human brain is not fixed: neural pathways are reshaped by deliberate practice and environmental input, and cognitive-behavioral strategies and the intentional cultivation of empathy have been proposed to strengthen prefrontal control over more impulsive, reward-driven systems [74,75]. In this sense, self-regulatory capacity is less a matter of independence from biology than of how that capacity has been built or eroded over time. Genetic variation may influence how strongly an impulse is felt; experience and deliberate effort influence what is done with it. No study has examined whether such strategies alter infidelity-related behavior; the neuroplasticity evidence cited concerns stress regulation and self-control more broadly [74,75].

## 7. Discussion

The evidence reviewed here supports a genetic contribution to individual differences in extrapair mating, but it does not identify a reproducible set of variants that can predict infidelity. The Finnish study estimated broad-sense heritability at 62% in men and 40% in women, describing population-level variance in a specific context [23]. The only other twin estimate, obtained in UK female twin pairs, is lower and was derived from a different phenotype definition [22].

The molecular evidence is less consistent than the heritability evidence. Almost all of the behavioral findings reviewed were derived from single-cohort candidate gene studies with small sample sizes. The *DRD4* finding is notable because it directly addressed sexual infidelity; however, it came from a small sample, and the categorical association with infidelity in that sample was not statistically significant [27]. This result is hypothesis-generating rather than evidence of a causal pathway. For *DAT1*, a large-sample study reported an association with the number of sexual partners, which remains an adjacent rather than a direct phenotype [43].

The *OXTR* literature highlights the importance of phenotype definition and replication. One study measured responses to a laboratory breach of trust in a game rather than in a relationship [47]. Other studies examined pair bonding or marital satisfaction and reported a potential association between certain *OXTR* SNPs and the phenotype [44,53]. Meta-analytic evidence for *OXTR* rs53576 did not detect an association with close relationships [54,55]. AVPR1A findings are similarly inconsistent, with associations reported in some samples but not others [22,59,60].

Multiple testing further limits interpretation. Several of the studies reviewed tested many variants, haplotypes, and outcomes in a single sample, and fewer associations survived formal correction than were reported at nominal significance. In the *OXTR* trust-violation study, one of the three reported haplotype associations was significant after correction [47]. In the *AVPR1A* pair bonding study, one of the eleven RS3 alleles tested remained associated with partner bonding after Bonferroni correction [59].

*MAO-A* is interpreted as an indirect candidate related to behavioral regulation. Its strongest evidence concerns impulsivity, aggression, and gene–environment interaction with childhood maltreatment [67]. The broader methodological literature indicates that candidate gene-by-environment findings in other domains, such as psychiatry, are consistent with publication bias and low power [19], and that favored candidate genes have failed when tested at scale in those domains [20]. These concerns are particularly relevant to the genetic study of infidelity, where the phenotype is socially sensitive, typically self-reported, and inconsistently defined across studies.

Phenotypic heterogeneity is an important limitation in interpreting the genetic literature on betrayal, because the studies included under this broad concept do not necessarily examine the same behavioral outcome. Infidelity or extrapair mating, pair–bond formation and relationship stability, emotional or behavioral responses to a breach of trust, and traits such as novelty seeking, impulsivity, and aggression represent related but distinct phenotypes. For example, having multiple sexual partners does not necessarily indicate infidelity, just as relationship dissatisfaction does not necessarily indicate a breach of exclusivity. Combining these outcomes as though they were interchangeable may give the impression of greater consistency across studies than the evidence actually supports.

Ancestry and population specificity are further limitations. Nearly all of the primary evidence came from Sweden, the United Kingdom, and the United States, in samples of predominantly European ancestry. Allele frequencies at several of these loci may differ across populations. Because heritability and genotype–phenotype associations are properties of a population in an environment rather than of a genotype in isolation [24], neither the Finnish heritability estimate nor the candidate gene associations can be assumed to transfer to other populations, and no predictive application could responsibly be contemplated on the basis of the present evidence. Latin American, African, Asian and admixed populations, in which local ancestry can be modeled, are essentially unrepresented in this literature.

Sex differences in heritability and in gene effects deserve attention. Broad-sense heritability of recent extrapair mating was higher in men than in women in the Finnish sample [23]. These differences may reflect evolutionary divergence in the adaptive significance of extrapair mating for each sex, differential willingness to disclose the behavior, or limitations of the studies themselves. However, the same study found no between-sex genetic correlation, which argues against the hypothesis that a propensity to extrapair mating in women is a by-product of selection acting on men, and is consistent with at least partly distinct genetic and environmental influences in each sex [23].

## 8. Conclusions

The evidence does not support the idea that humans are genetically programmed to be unfaithful, but it does support the possibility that genetic variation contributes to individual differences in traits and processes that can influence relationship behavior. Dopaminergic, oxytocinergic, vasopressinergic, and monoaminergic systems are biologically relevant to reward, attachment, social behavior, and impulse control, but associations between particular variants and betrayal-related phenotypes remain small, heterogeneous, and incompletely replicated.

Extrapair mating is heritable at the population level, although the two available estimates differ in magnitude and neither has been reproduced in a comparable sample. Moreover, the molecular functions of the five loci reviewed are well characterized: the variants act on receptor density, transporter density, or enzymatic output, and the recurring mechanism is dosage rather than loss of function. Lastly, no variant reviewed here has demonstrated a replicable association with infidelity itself, and the two most intensively studied oxytocinergic polymorphisms show no aggregate association with close relationships. The integrated pathway model, the direction of several molecular effects in the living human brain, and any inference from genotype to an individual’s relational conduct remain unresolved.

Genes are only one part of the explanation. Experiences, relationship dynamics, cultural expectations, and cognitive control shape how biological tendencies are expressed. Therefore, the candidate genes discussed here do not determine betrayal, although they may contribute to differences in probability within particular contexts. Understanding these contributions may eventually clarify why individuals respond differently to temptation, attachment, conflict, or betrayal, without reducing complex human choices to a single genetic explanation. Priorities for the field are harmonized phenotype definitions, adequately powered genome-wide designs, ancestrally diverse and couple-level sampling, and functional validation of each variant before a behavioral meaning is assigned to it.

## 9. Future Perspectives and Knowledge Gaps

Future molecular studies should move beyond isolated candidate variants toward adequately powered genome-wide approaches and functional follow-up. Existing genome-wide data address adjacent behaviors and indicate a highly polygenic architecture with per-variant effects far smaller than those reported in candidate gene work [18,25,26]. Harmonized phenotype definition is a precondition, since inconsistent measurement of infidelity across cohorts cannot be offset by sample size alone.

Functional validation studies should proceed in parallel. For each gene variant, the molecular effect should be tested at the cellular level. The claims that the *AVPR1A* RS3 repeat alters promoter activity [61], that the *DRD4* exon III VNTR alters G-protein coupling efficiency [30,32], and that the *MAO-A* uVNTR sets transcriptional output [64,65] each require confirmation in the relevant cell types. Given the discordance already documented for *DAT1* between in vitro and in vivo assays [40,42], such work should report results at both levels rather than generalizing from one to the other.

Epigenetic mechanisms, particularly DNA methylation at social bonding and stress regulation loci, are a plausible route by which early-life experience, chronic stress, and caregiving environments could shape later relational behavior without altering DNA sequence, and OXTR is a reasonable starting point because methylation at this locus has been linked to receptor availability [51,71]. Epigenetic association studies are subject to the same power and tissue-specificity problems as candidate gene work.

Greater population diversity is essential, because allele frequencies at these loci vary across ancestries and because the social environments that moderate relational behavior differ substantially. Studies that genotype both members of a couple are a further priority, since betrayal occurs within a relationship rather than within an individual, and such designs would also allow the relational-phase hypothesis raised by the discordant AVPR1A findings to be tested directly [59,60]. Finally, the field would advance through longitudinal, genome-wide, and multilevel studies that integrate genetics with epigenetics, neurobiology, psychology, and relationship context. Functional validation will be essential for determining whether associated variants alter gene expression, receptor function, or neural phenotypes in ways relevant to behavior. The goal should not be to identify a “gene for infidelity,” but to understand how many small biological influences interact with experience and social context to shape human relationships.

## Figures and Tables

**Figure 1:**
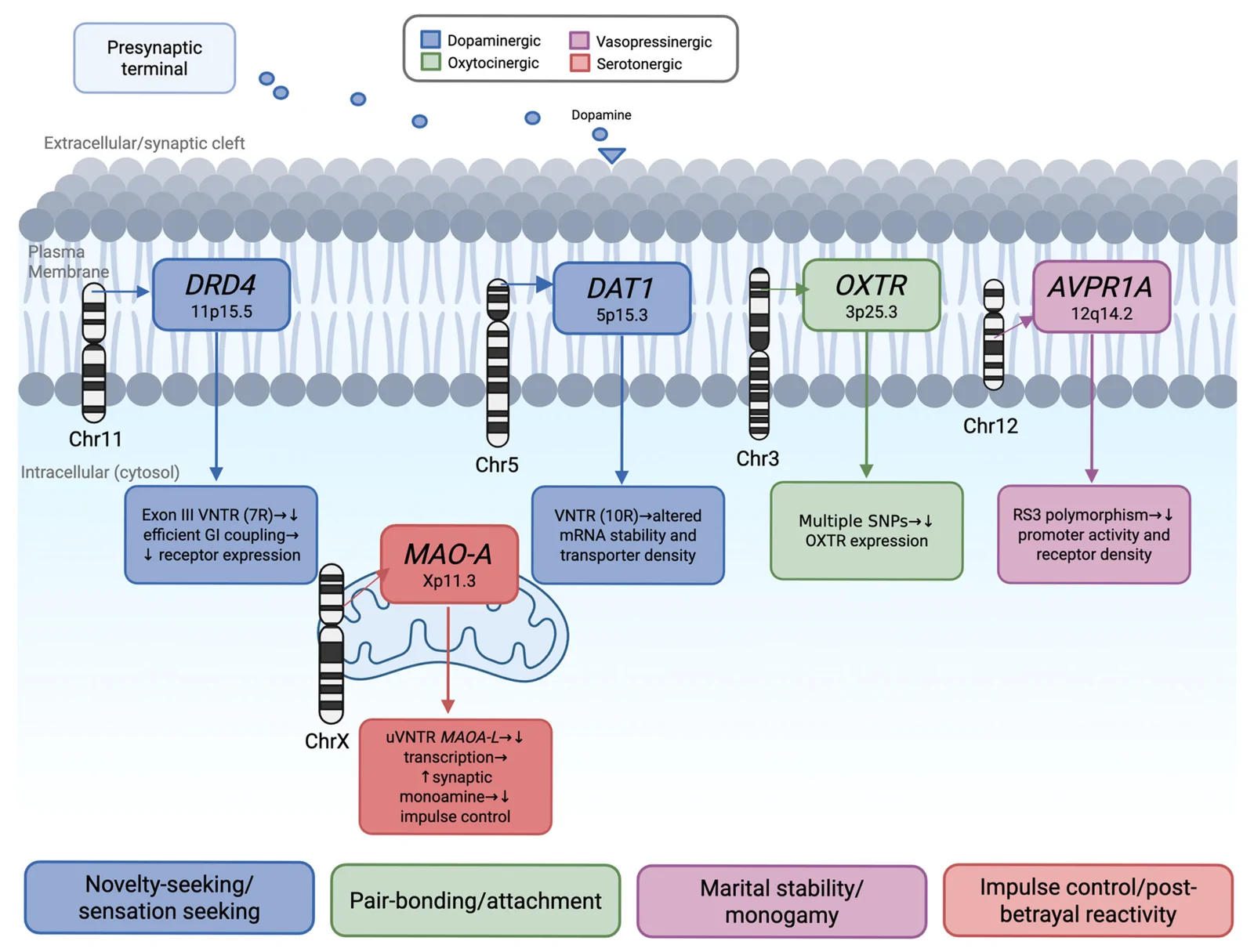
Proposed relationships between candidate gene variation, neurotransmitter system tone, and betrayal-related behavior. Five candidate genes and their chromosomal locations are shown (*DRD4*, *DAT1*, *AVPR1A*, *OXTR*, and *MAO-A*). Each row shows the gene variant and the pathway modification. Each gene variant is proposed to modulate the functional tone of its respective neurotransmitter system and, through it, the behavioral outputs shown. Created in BioRender. Zambrano A.K. (2026). https://BioRender.com/dh1r3kr, accessed on 23 June 2026. *Abbreviations:* Chr, chromosome; VNTR, variable number tandem repeat; *DAT1*, dopamine transporter gene (SLC6A3); *DRD4*, dopamine receptor D4 gene; *AVPR1A*, arginine vasopressin receptor 1A; *OXTR*, oxytocin receptor gene; *MAO-A*, monoamine oxidase A. The downward arrows (↓) indicate a decrease, whereas the upward arrows (↑) indicate an increase. The large arrows connect the gene with the corresponding gene variant and its possible associated pathway modification.

**Table 1:** Evidence matrix summarizing candidate genes, chromosomal location, protein products, signaling pathways, variants, associated effects, and proximity of the reported phenotype to infidelity.

Gene	Chromosomal Location	Protein Product and Coupling/Cofactor	Signaling Pathway/Messenger	Variant(s)	Associated Effect and Phenotype Proximity	Source
*DRD4*	11p15.5	G-protein-coupled dopamine receptor	↓ adenylyl cyclase → ↓ cAMP → ↓ PKA	48 bp VNTR, exon III (≥7 repeats)	Sensation seeking (category iv), disinhibition and sexual infidelity (category i).	[27,30,34,35,36,37,38]
*DAT1/SLC6A3*	5p15.3	Na^+^/Cl^−^-dependent dopamine transporter	Presynaptic dopamine reuptake; sets transmitter clearance	40 bp VNTR, 3’UTR (10-repeat)	Number of sexual partners (category iv).	[40,42,43,70]
*OXTR*	3p25.3	Oxytocin receptor	Gαq/11 → PLC-β → IP_3_/Ca^2+^ and DAG/PKC; ERK/β-arrestin	Multiple SNPs, including rs9840864, rs2268494, rs237887, rs2268490, rs7632287 and rs53576	Pair bonding (category ii), speed of retaliation after betrayal (category iii), and marital satisfaction (category ii).	[44,47,52,53,54,55,56,57]
*AVPR1A*	12q14.2	Vasopressin V1a receptor	Gαq/11 → PLC → Ca^2+^/PKC	RS3 polymorphism (allele 334)	Influences pair bonding, monogamy, and marital satisfaction (category ii).	[22,23,59,60,61,62]
*MAO-A*	Xp11.3	Flavoenzyme	Oxidative deamination of monoamines	30 bp promoter uVNTR (low- vs. high-activity)	Reduced impulse inhibition, heightened aggressive response (category iv)	[63,65,66,67,68,69]

cAMP: cyclic adenosine monophosphate; DAG: diacylglycerol; IP_3_: inositol trisphosphate; VNTR: variable number tandem repeat; PKA: protein kinase A; PKC: protein kinase C; PLC-β: phospholipase C-β; SNP: single nucleotide polymorphism. Phenotype categories (i to iv) are defined in the literature identification section (Section 2).

**Table 2:** Evidence table for the primary behavioral association studies discussed, showing study sample, phenotype measured, variant, principal finding, reported effect size, phenotype proximity to infidelity or betrayal, and replication status.

Study	Study Sample	Phenotype Measured	Variant	Principal Finding	Reported Effect Size	Proximity to Target Phenotype	Replication Status
Zietsch et al. 2015 [23]	7378 Finnish twins and siblings	Extrapair mating over one year	Twin modeling and *AVPR1A*, *OXTR*	Heritability of extrapair mating: 62% men and 40% women; *AVPR1A* associated in women only; *OXTR* not associated in either sex	Variance components; wide confidence intervals; additive and non-additive components not separable	Direct	Not replicated in other populations
Karlsson et al. 2019 [25]	Over one million individuals, UK Biobank, 23andMe	Risk tolerance and risky behavior (number of sexual partners)	GWAS	SNP-based heritability was 0.128 for number of sexual partners	Polygenic score predicted 0.8% of the variance in number of sexual partners	Adjacent	Replication cohorts within the same study
García et al. 2010 [27]	181 young adults from one US university (61% European ancestry)	Infidelity and number of sexual partners	*DRD4* exon III VNTR (7 repeats or more and fewer than 7 repeats)	Promiscuity (“one-night stand”) 45% vs. 24%, *p* = 0.018. More extrapair partners among those already unfaithful (1.79 vs. 1.14, *p* = 0.011)	χ^2^ = 5.58 (promiscuity); F = 7.08 (extrapair partners)	Direct	Not replicated in other populations
Guo et al. 2007 [43]	~ 2500 US young adults	Number of sexual partners	*DAT1* 3′UTR VNTR (10R/10R vs. 9R/9R)	More sexual partners in 10-repeat homozygotes; effect interacted with sex and social network	Not reported	Adjacent	Not replicated in other populations
Walum et al. 2012 [44]	2309 (Twin and Offspring Study in Sweden) and 1240 (Swedish Twin Study of Child and Adolescent Development) Swedish individuals	Pair bonding and social behavior	*OXTR* SNPs	Association between specific SNPs and pair-bonding-related measures	Not reported	Adjacent	Not replicated in other populations; rs7632287 independently associated with autism in meta-analysis [52].
Tabak et al. 2013 [47]	165 US university students	Retaliation and satisfaction after trust violation in an iterated Prisoner’s Dilemma against a pre-programmed computer partner	10 *OXTR* SNPs	Variants linked to retaliation speed and post-betrayal satisfaction	Not reported	Indirect (Laboratory analog)	Not replicated
Monin et al. 2019 [53]	178 US married couples	Attachment security and marital satisfaction	*OXTR* rs53576	GG genotype associated with more secure attachment and higher marital satisfaction	Not reported	Adjacent	Not replicated for the same phenotype; inconsistent with meta-analytic findings [54,55]
Bakermans-Kranenburg & van Ijzendoorn 2014 [54]	Meta-analysis with *n* = 17,559 for rs53576 and *n* = 13,547 for rs2254298	Biology, personality, social behavior, psychopathology, autism	*OXTR* rs53576, rs2254298	No association between the SNPs and the social behaviors analyzed	Combined effect sizes did not differ from zero in any domain	Indirect	Not replicated in other populations for both SNPs across all domains
Li et al. 2015 [55]	General sociality sample of 4955 and close relationship sample of 5262	General sociality and close relationship	*OXTR* rs53576	No significant association between the polymorphism and close relationships, but an association between the polymorphism and general sociality	General sociality d = 0.11 (95% CI 0.02–0.21, *p* = 0.02); close relationships d = 0.01 (95% CI −0.04 to 0.07, *p* = 0.64)	Adjacent	Not replicated in other populations
Scheele et al. 2013 [56]	20 men in the discovery study and 20 men in the replication study	Effect of intranasal oxytocin on perceived attractiveness of the partner’s face	Intranasal oxytocin	Enhanced reward-system response to the partner’s face relative to a stranger’s	Not reported	Adjacent	Not replicated in other populations; internal replication within the same study
Walum et al. 2008 [59]	552 Swedish twin pairs and their spouses	Partner bonding, marital crisis and marital status	*AVPR1A* RS3 allele 334	Male carriers reported lower marital satisfaction and more marital crisis	d = 0.27 (carriers vs. non-carriers); d = 0.38 (homozygotes); marital crisis 15% vs. 34%; spouse ratings d = 0.14–0.20	Adjacent	Direction reversed in [60]
Cherkas et al. 2004 [22].	Over 1600 UK female twin pairs	Infidelity and number of sexual partners	*AVPR1A*	No association with *AVPR1A*	Heritability 41% (infidelity) and 38% (number of partners); no *AVPR1A* association	Direct	Not replicated in other populations
Makhanova et al. 2025 [60]	70 US newlywed couples	Relationship maintenance over 3 years and interest in infidelity	*AVPR1A* RS3 allele 334	334 carriers reported fewer marital problems and less interest in romantic alternatives	Not reported	Adjacent	Contradicts [59]
Meyer-Lindenberg et al. 2006 [67]	Healthy adult volunteers	Amygdala and cingulate volume/activity; impulsive violence	*MAO-A* uVNTR (high vs. low activity)	Low-activity carriers showed volume reductions and, in men, impulsive violence	Not reported	Indirect	Not independently replicated

n: sample size; CI: confidence interval; GWAS: genome-wide association study; SNP: single-nucleotide polymorphism; d: Cohen’s standardized difference between groups; χ^2^: chi-square; F: F-statistic; *p*: *p*-value. Proximity to target phenotype: “direct” denotes measurement of infidelity or extrapair mating; “adjacent” denotes a related relationship or sexual behavior outcome; “indirect” denotes a trait such as impulsivity, aggression, or laboratory trust behavior that is not a measure of betrayal. Effect sizes are given only where the source study reported them.

## Data Availability

No new data were generated or analyzed as part of this study; therefore, there are no datasets available for sharing.
